# Awareness of the Importance of Zinc and the Effects of Its Deficiency Among the General Population of Jeddah, Saudi Arabia

**DOI:** 10.7759/cureus.76704

**Published:** 2024-12-31

**Authors:** Noha M Hazem, Aleya Alhusayni, Farihah Arbee, Swailma Hyder, Meimona Ennahoui, Jumana Alhasan, Wed Bamujally

**Affiliations:** 1 Pathological Sciences, Fakeeh College for Medical Sciences, Jeddah, SAU; 2 Clinical Sciences, Fakeeh College for Medical Sciences, Jeddah, SAU

**Keywords:** awareness, deficiency, importance, saudi arabia, zinc

## Abstract

In recent decades, zinc deficiencies have emerged as a significant concern in the global health field. Zinc is important for the optimal functionality of all the body systems. Its supplementation has gained considerable traction as an essential focus, notably during the COVID-19 pandemic. Our study aimed to assess the level of awareness and knowledge regarding zinc's importance, sources, and the causes and effects of zinc deficiency. Our cross-sectional study was conducted among the general population of Jeddah, Saudi Arabia, through a self-administered questionnaire. Ethical approval was obtained from the Fakeeh College Scientific Research Review Committee (Jeddah, SAU). The scoring system gives the participant 1 point for each correct answer and 0 points for every wrong answer, with the overall score being out of 20. Several demographic factors, including nationality, age, gender, healthcare status, place of residency, and marital status, were investigated among 384 participants. Upon analysis, the average overall knowledge score was estimated to be 15.67 out of 20. Significant differences were found in the knowledge scores between different genders and educational levels. It was thus concluded that these knowledge gaps need to be addressed by developing different targeted health campaign strategies and interventions to increase awareness in the population.

## Introduction

During the past decades, zinc deficiencies have been a major concern in the medical field with an estimated prevalence of 17.3% worldwide [1]. After discovering the first case of human zinc deficiency in 1961, new studies were performed exploring the importance of adequate zinc levels and the effects of its deficiency [2]. During the COVID-19 pandemic, several studies suggested that zinc supplementation enhances the immune system and reduces the mortality and illness duration of COVID-19 patients [3]. Therefore, during this time, the interest in zinc supplementation was at its peak around the world.

Zinc is an essential trace mineral that plays a critical role in the human body. It has been known to act as a coenzyme for over 300 enzymes, thus impacting cellular growth, cell differentiation, and the metabolism of lipids and proteins [4]. It is also important for immune system functionality, reproduction, fetal development, growth, and tissue repair [4]. Although zinc is considered one of the most abundant minerals in the body, it cannot be stored in large amounts, thus requiring regular adequate intake and absorption [5]. As with most micronutrients in the human body, zinc deficiency can be due to low intake, reduced absorption, increased utilization, or increased excretion, and the most common cause of zinc deficiency currently is due to reduced intake [5]. Without proper supplementation and care, zinc deficiency poses a great threat to the immune system, contributing to the development of impaired taste and smell sensations, growth impairment in children, intestinal flora alteration, atopic dermatitis, iron-deficiency anemia, as well as non-specific symptoms like diarrhea, brittle nails, and hair loss [5].

Zinc can be found in numerous food groups, including animal protein, fish, legumes, nuts, and other dietary sources [6]. Many of these components can be found in the Mediterranean diet; however, despite this, a recent study conducted in Saudi Arabia showed that 15.3% of the population had zinc deficiency [1]. It was also found that 64.3% had vitamin D deficiency, 44.8% had B12 deficiency, and 23.2% had iron deficiency [7]. The high prevalence of mineral deficiencies observed in this study underscores a significant lack of awareness regarding the importance of minerals and micronutrient deficiencies in Saudi Arabia [5]. This study therefore aims to assess the level of awareness regarding the importance of zinc and the impacts of its deficiency among the general population of Jeddah, Saudi Arabia, especially as research on zinc deficiency in the Kingdom is remarkably limited.

## Materials and methods

This cross-sectional study was conducted among the general population of Jeddah through a self-administered online questionnaire using Google Forms (Google LLC, Menlo Park, CA, USA) [8]. To calculate the required sample size for the population of Jeddah (which is estimated to be 4 million), a confidence level of 95% and a confidence interval of 5% were used, and thus the required sample size was determined to be 384 participants [9]. The questionnaire was designed by the authors after revising a previous similar study assessing Vitamin D awareness, which was validated through Cronbach’s alpha reliability score [10]. The designed questionnaire was then reviewed and validated by a clinical biochemist and a public health analyst. Ethical approval was obtained from the Scientific Research Review Committee at Fakeeh College of Medical Sciences, Jeddah, Saudi Arabia (approval No. 292/IRB/2022).

Before the main research, a pilot study was carried out to ensure the validity and reliability of the self-administered online questionnaire utilized in this study on zinc importance and the effects of its insufficiency among the general population of Jeddah, Saudi Arabia. The pilot study included a small sample of 30 individuals from diverse demographic categories to evaluate the questionnaire items' clarity, relevance, and efficacy. Based on the input, numerous changes were made to improve the questionnaire's readability and comprehensiveness. Ambiguous questions were rephrased for more clarity, and response options were improved to ensure that the required data was captured correctly. In addition, professional input was sought to assess the questionnaire's content and format, ensuring that it was consistent with the research aims and included all key areas of zinc awareness.

All participants received information about the aim of the study, and clear consent was obtained from them. Participants eligible for inclusion in this study were male and female residents of Jeddah who were 18 years of age or older. These individuals were part of the general population, ensuring a diverse and representative sample of adults within the community. The study included individuals who were capable of providing informed consent and willing to participate in a survey regarding their awareness of the importance of zinc and the potential for zinc deficiency. Individuals who are under the age of 18 were automatically excluded.

Individuals who were unable to provide informed consent or who were unwilling to participate in the survey were also excluded and not allowed to complete the survey. This ensured that all participants were voluntarily able to comprehend the significance of the research topic. The questionnaire consisted of six sections with 28 questions in total, all translated into English (see Appendix A) and Arabic [11]. The first section explained the aim of the study and took consent from the participants. The second section collected demographic information, including gender, age, educational level, residential area, marital status, and whether the participant was a healthcare worker/student. Section three assessed the awareness regarding the importance of zinc. Section four assessed the awareness regarding the sources of zinc. Section five assessed the awareness regarding the causes of zinc deficiency. The last section assessed the awareness regarding the effects of zinc deficiency. All information used in the questionnaire questions was taken from reliable sources with up-to-date evidence [12-19]. The scoring system used gave the participant 1 point for each correct answer and 0 points for wrong answers with an overall score of 20. The SPSS Statistics version 29 (IBM Corp., Armonk, NY, USA) [20] and Microsoft Excel 2021 (Microsoft Corp., Redmond, WA, USA) were used to statistically analyze the collected data to interpret results and draw a conclusion. All non-parametric data were expressed as numbers and percentages, whereas parametric data were expressed as means and standard deviations. Participants’ knowledge of zinc and its deficiency was compared amongst the demographic groups using an independent t-test and a one-way variance analysis test. A p-value of less than 0.05 was considered statistically significant.

## Results

The link to the questionnaire was shared randomly among the population through text messages as well as social media. Out of the 384 participants, 252 (65.6%) were between the ages of 18 and 25, 243 (63.3%) were Saudi, 291 (75.8%) were females, 198 (51.6%) had an undergraduate degree, 122 (31.8%) had completed high school, 196 (51.0%) were healthcare workers/students, and 188 (49.0%) were non-healthcare workers, 260 (67.7%) were single, 141 (36.7%) lived in the north of Jeddah, and 113 (29.4%) lived in the center of Jeddah (Table 1).

**Table 1:** Demographic data of the participants

Variables	N	%
Age		
18-25	252	65.6%
26-35	53	13.8%
36-45	37	9.6%
46-55	33	8.6%
56 and older	9	2.3%
Nationality		
Saudi	243	63.3%
Non-Saudi	141	36.7%
Gender		
Males	93	75.8%
Females	291	24.2%
Level of Education		
High school degree	122	31.8%
Undergraduate degree	198	51.6%
Postgraduate degree	50	13.0%
Diploma	14	3.6%
Healthcare worker/student		
Yes	196	51.0%
No	188	49.0%
Marital status		
Single	260	67.7%
Married	112	29.2%
Divorced	9	2.3%
Widow	3	0.8%
Place of residency		
North of Jeddah	141	36.7%
West of Jeddah	47	12.2%
East of Jeddah	42	10.9%
South of Jeddah	41	10.7%
Center of Jeddah	113	29.4%

The level of awareness regarding the importance of zinc is shown in Table 2. The majority of the participants (95.1%) agreed that zinc is important for the immune system and gastrointestinal tract; 84.1% were aware that zinc is important for protein and lipid metabolism as well as gene transcription; and 93.0% knew that zinc is required for proper growth and development. On the other hand, 26.6% did not know that zinc supplementation in severely deficient patients can reduce their risk of mortality (Table 2).

**Table 2:** The level of awareness about the importance of zinc to different aspects of health among the general population of Jeddah

Variables	N	%
Zinc is important for the immune system and gastrointestinal tract		
True	365	95.1%
False	19	4.9%
Zinc is important for protein and lipid metabolism as well as gene transcription		
True	323	84.1%
False	61	15.9%
Zinc is a requirement for proper growth and development		
True	357	93.0%
False	27	7.0%
Zinc supplementation in severely deficient patients can reduce their risk of mortality		
True	282	73.4%
False	102	26.6%

The results assessing the awareness regarding the sources of zinc (Table 3) show that 44.5% of the population were not aware that nutrient-fortified breakfast cereals contain the second highest content of zinc, and 34.1% did not know that fruits and vegetables contain low levels of zinc. On the contrary, 91.7% of the participants understood that meats, fish, and seafood are known to contain the greatest content of zinc, 77.3% agreed that oysters have the highest content of zinc, and 81% knew that raw nuts contain a great amount of zinc (Table 3).

**Table 3:** Awareness about the different dietary sources of zinc

Variables	N	%
Meat, fish, and seafood are known to contain the greatest content of zinc		
True	352	91.7%
False	32	8.3%
Oysters have the highest content of zinc		
True	297	77.3%
False	87	22.7%
Nutrient-fortified breakfast cereals contain the second-highest content of zinc		
True	213	55.4%
False	171	44.5%
Fruits and vegetables contain low levels of zinc		
True	253	65.9%
False	131%	34.1%
Raw nuts contain a great amount of zinc		
True	311	81%
False	73	19%

Table 4 demonstrates the results assessing the awareness regarding the causes of zinc deficiency. Almost 80% of the participants agreed that vegetarians and vegans have a higher chance of developing zinc deficiency. Half the participants were not aware that patients taking antihypertensive medications are less prone to developing zinc deficiency. Around 84.6% were well aware that pregnant and lactating females are at a higher risk of developing zinc deficiency, and 89.6% knew that individuals with malabsorption regardless of the cause are at a higher risk of developing zinc deficiency.

**Table 4:** Awareness about the varying causes that could lead to zinc deficiency

Variables	N	%
Vegetarians and vegans have a higher chance of developing zinc deficiency		
True	307	79.9%
False	77	20.1%
Individuals who take antihypertensive medications are less prone to developing zinc deficiency		
True	190	49.5%
False	194	50.5%
Pregnant and lactating females are at a higher risk of developing zinc deficiency		
True	325	84.6%
False	59	15.4%
Individuals with malabsorption regardless of the cause are at a higher risk of developing zinc deficiency		
True	344	89.6%
False	37	9.6%

The last portion of the survey assessed the awareness regarding the effects of zinc deficiency as shown in Table 5. Around 84.9% of the participants agreed that zinc deficiency can cause both immune dysfunction and increase the risk of inflammatory and autoimmune diseases as well as growth impairment in children. The fact that zinc deficiency could cause non-specific symptoms such as diarrhea, hair loss, and brittle nails was known by 87% of the participants; 63.0% of the participants agreed that zinc deficiency causes impaired taste and smell sensations. An average of 78.6% of the population agreed that zinc deficiency enhances intestinal inflammation and alters the intestinal flora and that zinc deficiency could be a cause of iron deficiency anemia. Lastly, 68.8% of the participants chose 'true' for zinc deficiency being a contributing cause of atopic dermatitis.

**Table 5:** The level of awareness about the effect of zinc deficiency on health

Variables	N	%
Zinc deficiency causes impaired taste and smell sensations		
True	242	63.0%
False	142	37.0%
Zinc deficiency enhances intestinal inflammation and alters the intestinal flora		
True	304	79.2%
False	80	20.8%
Zinc deficiency can be a contributing cause of atopic dermatitis		
True	264	68.8%
False	120	31.3%
Zinc deficiency can cause growth impairments in children		
True	326	84.9%
False	58	15.1%
Zinc deficiency can cause immune dysfunction and increase the risk of inflammatory and autoimmune diseases		
True	326	84.9%
False	58	15.1%
Zinc deficiency could be a cause of iron deficiency anemia		
True	300	78.1%
False	84	21.9%
Non-specific symptoms of zinc deficiency include diarrhea, hair loss, and brittle nails		
True	334	87%
False	50	13%

The average overall knowledge score for all participants was estimated to be 15.67 ± 3.29 out of 20. The percentage of true answers for the questions assessing the importance of zinc was 86.5%, 74.3% for the sources of zinc, 76.1% for the causes of its deficiency, and 78% for the effects of deficiency, making the overall percentage of true answers 78.3%. The percentage of total false answers was 21.7% (Table 6). When comparing Saudi participants with non-Saudi participants, there was no significant difference found for all knowledge domains (importance of zinc, sources of zinc, causes of zinc deficiency, and effect of zinc deficiency) (Table 7).

**Table 6:** Average overall knowledge scores for the importance of zinc, sources of zinc, cause of zinc deficiency, and effects of zinc deficiency

Variables	Mean ± SD	Range (min-max)	Total percentage
Importance of zinc	3.46 ± 0.80	0-4	86.5%
Sources of zinc	3.71± 1.06	0-5	74.3%
Cause of zinc deficiency	3.04 ± 0.89	0-4	76.1%
Effects of zinc deficiency	5.46 ± 1.57	0-7	78%
Overall	15.67 ± 3.29	0-20	78.3%

**Table 7:** Knowledge scores by nationality on the importance of zinc, its sources, and the causes of deficiency and its effects *A p-value < 0.05 is significant.

Variables (nationality)	Mean ± SD	p-value
Importance of zinc		
Saudi	3.50 ± 0.77	0.19
Non-Saudi	3.38 ± 0.86	
Sources of zinc		
Saudi	3.77 ± 1.04	0.22
Non-Saudi	3.62 ± 1.09	
Causes of zinc deficiency		
Saudi	3.05 ± 0.88	0.63
Non-Saudi	3.01 ± 0.91	
Effects of zinc deficiency		
Saudi	5.50 ± 1.51	0.76
Non-Saudi	5.43 ± 1.67	

When analyzing the knowledge scores among different age groups, there were no significant differences in the scores across all four knowledge domains (Table 8). However, it was noted that older age groups typically displayed better knowledge, with the age group of 56 years and higher displaying the greatest knowledge score.

**Table 8:** Knowledge scores by age group on the importance of zinc, its sources, and the causes of its deficiency and its effect *A p-value < 0.05 is significant.

Variables (age in years)	Mean ± SD	p-value
Importance of zinc		
18-25	3.48 ± 0.78	0.74
26-35	3.43 ± 0.80	
36-45	3.38 ± 0.79	
46-55	3.33 ± 1.02	
56 and older	3.67 ± 0.71	
Sources of zinc		
18-25	3.77 ± 1.07	0.21
26-35	3.47 ± 1.17	
36-45	3.68 ± 0.91	
46-55	3.61 ± 1.00	
56 and older	4.22 ± 0.97	
Causes of deficiency		
18-25	3.09± 0.89	0.26
26-35	2.92 ± 0.94	
36-45	3.05 ± 0.78	
46-55	2.76 ± 1.00	
56 and older	3.22 ± 0.67	
Effects of deficiency		
18-25	5.50 ± 1.53	0.09
26-35	5.11 ± 1.69	
36-45	5.59 ± 1.30	
46-55	5.24 ± 1.89	
56 and older	6.56 ± 1.01	

A slight gender difference was notable; while female participants generally demonstrated higher awareness than male respondents, there was only a significant difference between the two genders regarding their knowledge of the sources of zinc, with a significant p-value of 0.02. All other knowledge domains between the genders were insignificant (Table 9).

**Table 9:** Knowledge scores by gender on the importance of zinc, its sources, and the causes of deficiency and its effects *A p-value < 0.05 is significant.

Variables (gender)	Mean ± SD	p-value
Importance of zinc		
Males	3.37 ± 0.92	0.21
Females	3.48 ± 0.76	
Sources of zinc		
Males	3.49 ± 1.20	0.02*
Females	3.78 ± 1.01	
Causes of deficiency		
Males	2.97 ± 0.88	0.39
Females	3.06 ± 0.90	
Effects of deficiency		
Males	5.37 ± 1.73	0.51
Females	5.49 ± 1.51	

There were statistically significant differences in knowledge scores regarding the importance of zinc, sources of zinc, and effects of zinc deficiency among individuals with different education levels, with p-values of 0.0009, 0.0003, and 0.004, respectively. However, there were no significant differences in knowledge scores regarding the causes of deficiency amongst the different education levels (Table 10). It was found that participants possessing undergraduate and postgraduate degrees displayed overall higher knowledge scores across the four knowledge domains, in contrast to those with high school degrees and diplomas.

**Table 10:** Knowledge scores by level of education on the importance of zinc, its sources, and causes of deficiency and its effects *A p-value < 0.05 is significant.

Variables (education level)	Mean ± SD	p-value
Importance of zinc		
High school degree	3.33 ± 0.89	0.0009*
Diploma	2.86 ± 1.23	
Undergraduate degree	3.56 ± 0.68	
Postgraduate degree	3.54 ± 0.79	
Sources of zinc		
High school degree	3.62 ± 1.09	0.0003*
Diploma	3.36 ± 1.45	
Undergraduate degree	3.84 ± 0.97	
Postgraduate degree	3.54 ± 1.20	
Causes of deficiency		
High school degree	2.96 ± 0.90	0.23
Diploma	2.86 ± 1.03	
Undergraduate degree	3.14 ± 0.86	
Postgraduate degree	2.88 ± 0.94	
Effects of deficiency		
High school degree	5.26 ± 1.58	0.004*
Diploma	4.21 ± 2.01	
Undergraduate degree	5.62 ± 1.51	
Postgraduate degree	5.66 ± 1.47	

When assessing the knowledge of healthcare workers/students and those who weren’t, significant differences were found in the knowledge scores regarding the importance of zinc and the causes of deficiency. The former demonstrated higher awareness than the latter (Table 11).

**Table 11:** Knowledge scores per healthcare workers/students on the importance of zinc, its sources, and the causes of deficiency and its effects *A p-value < 0.05 is significant.

Variables	Mean ± SD	p-value
Importance of zinc		
Yes	3.55 ± 0.73	0.02*
No	3.36 ± 0.86	
Sources of zinc		
Yes	3.82 ± 1.05	0.05
No	3.61 ± 1.07	
Causes of deficiency		
Yes	3.16 ± 0.85	0.006*
No	2.91 ± 0.92	
Effects of deficiency		
Yes	5.56 ± 1.57	0.24
No	5.36 ± 1.57	

Marital status did not seem to have a significant impact on the awareness of zinc's importance, its sources, and causes of deficiency. While there may be a slight difference in the knowledge of the effects of deficiency, it is not statistically significant (Table 12). When comparing the knowledge scores of participants across different geographical regions in Jeddah, there was no statistical significance found between them.

**Table 12:** Knowledge scores by marital status on zinc's importance, its sources, and cause of deficiency and its effects *A p-value < 0.05 is significant.

Variables (marital status)	Mean ± SD	p-value
Importance of zinc		
Single	2.96 ± 0.67	0.83
Married	3.01 ± 0.61	
Divorced	2.84 ± 0.71	
Widow	3.00 ± 1.05	
Sources of zinc		
Single	3.05 ± 0.74	0.84
Married	3.07 ± 0.63	
Divorced	2.97 ± 0.55	
Widow	2.84 ± 0.43	
Causes of deficiency		
Single	2.94 ± 0.76	0.96
Married	2.88 ± 0.61	
Divorced	2.84 ± 0.76	
Widow	2.85 ± 0.58	
Effects of deficiency		
Single	3.03 ± 0.64	0.19
Married	2.94 ± 0.49	
Divorced	3.17 ± 0.56	
Widow	3.40 ± 0.62	

## Discussion

In this cross-sectional study, several demographic factors, including nationality, age, gender, healthcare worker status, place of residency, and marital status, were investigated amongst 384 participants to determine their influence on awareness levels regarding the importance, sources, causes, and effects of zinc deficiency. The findings of the study showed that slightly more than two-thirds of the population possessed sufficient knowledge of zinc deficiency. This could be attributed to the timing of the study, conducted post-COVID-19 pandemic when interest in zinc supplementation was at its peak. Zinc supplementation was discovered to play an essential role in the reproduction of SARS during the preceding coronavirus pandemic and its aftermath by inhibiting the SARS CoV-1 RNA (in vitro) [21]. During the COVID-19 pandemic, zinc deficiency (hypozincemia) was linked to significant consequences such as ARDS and increased mortality. Some studies proposed investigating the impact of zinc supplementation in the prevention of COVID-19 infection, but no data on its effectiveness has been published [22]. It can be noted there are still major knowledge gaps that need to be addressed.

The results of this study highlight that there is a dire necessity for public health strategies that specifically target the demographics that exhibited weaker awareness of the importance of zinc. For example, it was found that education level plays a significant role in the overall knowledge score; hence it is vital to ensure that there are more targeted health campaigns that focus on enhancing the knowledge of people. Age also influenced zinc awareness, with older age groups typically exhibiting better knowledge. This suggests that accumulated health experiences or sustained educational impacts over time shape awareness, indicating the need for age-appropriate educational interventions. Zinc supplements may help in preventing diseases caused by zinc deficiency; more funding for studies aimed at mapping the prevalence of zinc deficiency is necessary. For best results, community-level education programs ought to be promoted before zinc supplementation programs. Through the media, people should be made aware of micronutrient deficiencies, including zinc insufficiency, and how to prevent them by eating a balanced diet [23].

The Jeddah Health Promotion Authority has achieved tremendous progress in improving public knowledge about health and nutrition through community campaigns, collaboration with healthcare practitioners, and social media outreach. However, based on the study's findings, more work is needed to close the knowledge gaps around zinc insufficiency. Future attempts should involve designing focused educational programs for specific demographics, such as young people and those with poor educational attainment. These efforts could focus on sharing information in schools, businesses, and community centers. Additionally, using digital channels to distribute age-appropriate and entertaining information helps ensure a wider reach.

The Authority should also work with healthcare institutions to incorporate zinc awareness into routine health checks and consultations. Investing in studies to map zinc deficiency prevalence in Jeddah and tracking the efficacy of these therapies will be critical for long-term success. Promoting community-level education initiatives and promoting a balanced diet as a preventive step against micronutrient deficiencies will help improve the population's nutritional status and general health.

Such targeted strategies are crucial for overcoming the barriers identified in different demographic groups and their critical role in human health. Zinc has an impact on several immune system components, including lymphocyte gene regulation and the skin's protective barrier. It is essential for the healthy growth and operation of natural killer cells and neutrophils, two types of cells that mediate nonspecific immunity. Zinc has an impact on several immune system components, including lymphocyte gene regulation and the skin's protective barrier. Natural killer cells and neutrophils, two types of cells that mediate nonspecific immunity, require zinc for proper development and operation [24]. This would ultimately lead to better health outcomes and enhancement of the nutritional status of the community.

## Conclusions

The study tested 384 individuals in Jeddah, Saudi Arabia, on their understanding of the importance of zinc, its sources, and the causes and effects of zinc insufficiency. The results revealed considerable variations in knowledge scores based on gender, age, and education level. To address these gaps, stakeholders such as the Jeddah Health Promotion Authority, educational institutions, and local healthcare professionals must adopt focused interventions. For example, public health initiatives should target younger people and those with lesser educational attainment by utilizing relatable and easily accessible educational resources. Healthcare providers can incorporate zinc awareness into patient education during consultations, and schools and colleges can include zinc-related health information in their curricula. Media outlets should be enlisted to distribute specific messages aimed at raising awareness among marginalized populations. These measures will ensure that the individual needs of various demographic groups are satisfied, improving the effectiveness of health promotion initiatives and the community's overall nutritional health.

## Appendices

Appendix A

Presented below is the questionnaire to assess the level of awareness of zinc importance and impacts of zinc deficiency among the general population of Jeddah, Saudi Arabia.

Consent

This study aims to assess the level of awareness of zinc importance and deficiency among the general population of Jeddah, Saudi Arabia. By filling out this questionnaire, you agree to participate in this study. The participant must be a resident of Jeddah and 18 years of age or older. The collected information will be used for research purposes without identifying the participant’s identity.

Please read the questions carefully and answer them truthfully. Your participation is highly appreciated and we would like to thank you in advance.

I agree to participate in this study:

A) Yes

B) No

Demographics

1. Age

A) 18-25

B) 26-35

C) 36-45

D) 46-55

E) 56 and older

2. Gender

A) Female

B) Male

3. Nationality

A) Saudi

B) Non-Saudi

4. Place of residency

A) North of Jeddah

B) East of Jeddah

C) South of Jeddah

D) West of Jeddah

E) Center of Jeddah

5. Level of education

A) High school degree

B) Diploma

C) Undergraduate degree

D) Postgraduate degree

6. Healthcare worker/student

A) Yes

B) No

7. Marital status

A) Single

B) Married

C) Divorced

D) Widow

Category A: Awareness Regarding the Importance of Zinc

1. Zinc is important for the immune system and gastrointestinal tract

A) True

B) False

2. Zinc is important for protein and lipid metabolism as well as gene transcription

A) True

B) False

3. Zinc is a requirement for proper growth and development

A) True

B) False

4. Zinc supplementation in severely deficient patients can reduce their risk of mortality.

A) True

B) False

Category B: Awareness Regarding the Sources of Zinc

1. Meats, fish, and seafood are known to contain the greatest content of zinc.

A) True

B) False

2. Oysters have the highest content of zinc.

A) True

B) False

3. Nutrient-fortified breakfast cereals contain the second-highest content of zinc

A) True

B) False

4. Fruits and vegetables contain low levels of zinc

A) True

B) False

5. Raw nuts contain a great amount of zinc

A) True

B) False

*Category C: Awareness Regarding the Causes of Zinc Deficienc*y

1. Vegetarians and vegans have a higher chance of developing zinc deficiency

A) True

B) False

2. Individuals who take antihypertensive medications are less prone to developing zinc deficiency

A) True

B) False

3. Pregnant and lactating females are at a higher risk of developing zinc deficiency 

A) True

B) False

4. Individuals with malabsorption regardless of the cause are at a higher risk of developing zinc deficiency

A) True

B) False

Category D: Awareness Regarding the Effects of Zinc Deficiency

1. Zinc deficiency causes impaired taste and smell sensations

A) True

B) False

2. Zinc deficiency enhances intestinal inflammation and alters the intestinal flora

A) True

B) False

3. Zinc deficiency can be a contributing cause of atopic dermatitis

A) True

B) False

4. Zinc deficiency can cause growth impairments in children

A) True

B) False

5. Zinc deficiency can cause immune dysfunction and increase the risk of inflammatory and autoimmune diseases

A) True

B) False

6. Zinc deficiency could be a cause of iron-deficiency anemia

A) True

B) False

7. Non-specific symptoms of zinc deficiency include diarrhea, hair loss, and brittle nails

A) True

B) False
